# Who Is Food Insecure? Implications for Targeted Recruitment and Outreach, National Health and Nutrition Examination Survey, 2005–2010

**DOI:** 10.5888/pcd13.160103

**Published:** 2016-10-13

**Authors:** Sandi L. Pruitt, Tammy Leonard, Lei Xuan, Richard Amory, Robin T. Higashi, Oanh Kieu Nguyen, Carla Pezzia, Stephanie Swales

**Affiliations:** Author Affiliations: Tammy Leonard, Carla Pezzia, Stephanie Swales, University of Dallas, Irving, Texas; Lei Xuan, Robin T. Higashi, Oanh Kieu Nguyen, University of Texas Southwestern Medical Center, Dallas, Texas; Richard Amory, The Hunger Center, North Texas Food Bank, Dallas, Texas.

## Abstract

**Introduction:**

Food insecurity is negatively associated with health; however, health needs may differ among people participating in food assistance programs. Our objectives were to characterize differences in health among people receiving different types of food assistance and summarize strategies for targeted recruitment and outreach of various food insecure populations.

**Methods:**

We examined health status, behaviors, and health care access associated with food insecurity and receipt of food assistance among US adults aged 20 years or older using data from participants (N = 16,934) of the National Health and Nutrition Examination Survey from 2005 through 2010.

**Results:**

Food insecurity affected 19.3% of US adults (95% confidence interval, 17.9%–20.7%). People who were food insecure reported poorer health and less health care access than those who were food secure (*P* < .001 for all). Among those who were food insecure, 58.0% received no assistance, 20.3% received only Supplemental Nutrition Assistance Program (SNAP) benefits, 9.7% received only food bank assistance, and 12.0% received both SNAP and food bank assistance. We observed an inverse relationship between receipt of food assistance and health and health behaviors among the food insecure. Receipt of both (SNAP and food bank assistance) was associated with the poorest health; receiving no assistance was associated with the best health. For example, functional limitations were twice as prevalent among people receiving both types of food assistance than among those receiving none.

**Conclusion:**

Receipt of food assistance is an overlooked factor associated with health and has the potential to shape future chronic disease prevention efforts among the food insecure.

## Introduction

Food insecurity, defined by the US Department of Agriculture as having inconsistent access to adequate food because of limited financial and other resources, affects nearly 1 in 6 US households (1). People who are food insecure experience poor physical and mental health and face significant unmet needs for chronic disease prevention for conditions such as diabetes and hypertension (2–7). However, limited guidance exists for researchers and practitioners on how best to identify and recruit people who are food insecure for the purpose of advancing chronic disease prevention research, policy, and intervention.

In the United States, one way to target food insecure populations is through public and private food assistance programs. Publicly funded programs such as the Supplemental Nutritional Assistance Program (SNAP, formally known as “food stamps”) and the Special Supplemental Nutrition Program for Women, Infants, and Children (WIC) provide funds for food and grocery items that can be obtained at various retailers. In contrast, most privately funded programs provide groceries or meals directly to clients through loosely connected and often ad hoc assortments of nonprofit organizations. Programs have varying eligibility criteria; consequently, there is tremendous variation in use of both public and private food assistance among people who are food insecure (8).

Most previous studies on the health of food insecure populations recruited participants from a single food assistance program (eg, a WIC office, a food pantry) (9–12). This practice limits our knowledge as to whether health status varies among people participating in public and private programs. This practice also hampers our ability to understand whether recruitment and outreach strategies are equally effective among different subpopulations of people who are food insecure. Secondary analyses of survey data and other population-based studies (4,13) contribute to this knowledge gap by failing to stratify participation in food assistance programs. Therefore, people who are food insecure may be inadvertently characterized as belonging to a monolithic population with homogeneous patterns of health care use, health status, and food assistance. By obscuring distinct subpopulations facing unique health challenges, this approach handicaps the targeted design and delivery of effective chronic disease prevention and management interventions. Furthermore, many studies only assess a single outcome (eg, anemia, blood glucose) or behavior (eg, urgent care access, medication underuse) (7,13) and are often restricted to patients with chronic diseases (eg, people with diabetes) (4,14). A more comprehensive picture is needed of the overall spectrum of physical and mental health across various subpopulations of people who are food insecure.

The purpose of our study was to address these existing gaps in the literature. Specifically, we answered the following 3 questions: 1) what sociodemographic and health characteristics are associated with food insecurity among a national sample of US adults; 2) how do these sociodemographic and health characteristics differ across subpopulations of people who are food insecure, defined as participation in food assistance programs; and 3) what strategies can facilitate recruitment of food insecure populations receiving different types of food assistance. By elucidating health disparities within food insecure populations and reviewing competing strategies to recruit subpopulations of people who are food insecure, findings will inform future prevention research and interventions among this disadvantaged population.

## Methods

### Data

We conducted a cross-sectional analysis of nationally representative data from 16,934 respondents to the 2005 through 2010 US National Health and Nutrition Examination Survey (NHANES). Details on NHANES sampling strategy and data collection protocols are available elsewhere (15). We selected people who 1) were aged 20 years or older, the age at which education, a key covariate, is first assessed; and 2) answered all questions about food insecurity and food assistance. Institutional review board approval was not required because we used de-identified, publicly available data.

### Measures

**Food insecurity.** We ascertained household-level food insecurity by using the 18-item US Department of Agriculture Household Food Security Survey Module, which measures food insecurity for households with and without children (16). The scoring algorithm was provided in NHANES, and we categorized respondents as food secure or insecure (any degree of food insecurity including marginal insecurity), per recommendations and the growing understanding that any degree of food insecurity places people at risk for suboptimal health (3).

**Food assistance.** Two yes/no questions assessed whether, in the last 12 months, any household member received 1) SNAP (ie, public assistance) or 2) emergency food assistance from churches, food pantries, food banks, or soup kitchens (ie, charity or private assistance). We classified food-insecure households across 4 categories: no food assistance, SNAP only, charity only, and both SNAP and charity, according to previous research (8).

**Correlates. **We examined previous sociodemographic correlates of food security (4): age, sex, race/ethnicity, marital status, education, household size, number of children in the household aged 18 years or younger, and ratio of family income to the poverty threshold (PIR), which is calculated for each year in NHANES. We categorized PIR as less than 1, 1 to 1.32, 1.33 to 1.49, 1.50 to 1.84, and 1.85 or higher to reflect federal eligibility criteria for food and medical assistance (eg, Medicaid, free/reduced school lunch, WIC). A PIR of 1 or lower indicates income below the poverty level; thus, smaller PIRs indicate higher poverty and larger PIRs indicate lower poverty (Table 1).

**Table 1:** Sociodemographic and Health Characteristics of Food Secure Versus Food Insecure Populations, National Health and Nutrition Examination Survey, 2005–2010^a^

Characteristic	Total, N = 16,934	Food Secure, N = 12,379	Food Insecure, N = 4,555	*P* Value^b^
**Sociodemographics **				
**Age, y, mean (95% CI)**	46.8 (46.2–47.5)	48.2 (47.4–48.9)	41.1 (40.4–41.8)	<.001
**Sex**				
Male	48.1(47.5–48.7)	48.6 (48.0–49.2)	46.1 (44.6–47.7)	.005
Female	51.9 (51.3–52.5)	51.4 (50.8–52.0)	53.9 (52.3–55.5)	
**Race/ethnicity**				
Non-Hispanic white	69.9 (66.1–73.6)	75.6 (72.5–78.7)	46.0 (39.7–52.4)	<.001
Hispanic	12.6 (10.1–15.0)	8.9 (7.2–10.5)	28.1 (22.6–33.6)	
Non-Hispanic black	11.4 (9.6–13.2)	9.4 (7.8–11.0)	19.7 (16.2–23.3)	
Other	6.2 (5.1–7.3)	6.2 (5.0–7.3)	6.2 (4.6–7.8)	
**Marital status**				
Married	64.0 (62.5–65.5)	66.5 (65.0–68.0)	53.6 (51.3–55.9)	<.001
Single	17.2 (15.9–18.5)	15.8 (14.5–17.2)	23.0 (21.0–24.9)	
Widowed, separated, divorced	18.8 (18.0–19.6)	17.7 (16.9–18.5)	23.5 (21.8–25.2)	
**Education**				
Less than high school	19.0 (17.6–20.4)	14.9 (13.4–16.4)	36.3 (34.1–38.4)	<.001
High school graduate	24.3 (23.0–25.6)	23.4 (21.9–24.9)	28.0 (26.4–29.7)	
Some college, college degree, or more/other	56.6 (54.4–58.8)	61.6 (59.0–64.2)	35.5 (33.4–37.7)	
**Poverty-to-income ratio**				
<1	18.4 (17.0–19.7)	13.4 (12.3–14.6)	39.2 (36.5–41.9)	<.001
≥1 to <1.33	6.8 (6.1–7.4)	4.7 (4.0–5.4)	15.5 (13.9–17.1)	
≥1.33 to <1.50	3.4 (3.1–3.8)	2.8 (2.4–3.2)	6.1 (5.2–7.1)	
≥1.50 to <1.85	5.9 (5.4–6.5)	5.2 (4.5–5.9)	9.0 (7.8–10.2)	
≥1.85	65.5 (63.6–67.4)	73.9 (72.3–75.5)	30.1 (27.3–33.0)	
**Household size**				
1 or 2	47.5 (45.5–49.5)	50.9 (48.7–53.1)	33.3 (30.0–36.5)	<.001
3 or 4	35.9 (34.6–37.3)	35.4 (33.9–36.9)	38.2 (35.5–40.8)	
≥5	16.6 (15.0–18.2)	13.7 (12.2–15.3)	28.6 (25.2–32.0)	
**Children in home aged 18 y or younger**	40.8 (39.0–42.5)	37.0 (34.8–39.2)	56.5 (53.3–59.7)	<.001
**Health**				
**No. of days per month^c^**				
Poor physical health	3.6 (3.4–3.8)	3.2 (3.0–3.5)	5.1 (4.7–5.5)	<.001
Poor mental health	4.0 (3.8–4.2)	3.4 (3.2–3.6)	6.6 (6.1–7.0)	<.001
**Self-rated health^c^**				
Excellent/very good/good	75.6 (74.5–76.8)	78.8 (77.5–80.0)	62.6 (60.7–64.4)	<.001
Fair/poor	15.5 (14.5–16.5)	12.6 (11.6–13.6)	27.5 (25.7–29.2)	
**Functional limitations**				
Any	26.1 (24.6–27.6)	24.7 (23.1–26.4)	32.1 (29.8–34.3)	<.001
Activities of daily living	9.7 (9.1–10.4)	8.2 (7.5–8.9)	16.4 (14.9–17.9)	<.001
Instrumental activities of daily living	11.5 (10.8–12.3)	9.7 (8.9–10.5)	19.2 (17.7–20.6)	<.001
Lower extremity mobility	8.5 (7.8–9.2)	7.8 (7.1–8.6)	11.3 (10.0–12.7)	<.001
General physical activity	24.5 (23.0–25.9)	23.2 (21.6–24.8)	29.8 (27.6–32.1)	<.001
Leisure and social activities	9.1 (8.5–9.6)	7.4 (6.8–8.0)	16.0 (14.5–17.6)	<.001
**Comorbid conditions**				
Arthritis	24.6 (23.3–25.9)	25.0 (23.7–26.4)	23.0 (21.0–24.9)	.03
Cancer	9.1 (8.4–9.7)	9.9 (9.2–10.6)	5.6 (4.6–6.5)	<.001
Diabetes	8.2 (7.6–8.9)	8.0 (7.3–8.7)	9.3 (8.2–10.3)	<.04
Hypertension	30.0 (28.7–31.3)	30.5 (28.9–32.0)	28.0 (26.4–29.6)	.03
**Depressive symptoms in the last 2 weeks**				
None	80.4 (79.3–81.5)	83.6 (82.5–84.6)	67.4 (65.5–69.2)	<.001
Mild	16.7 (15.8–17.6)	14.6 (13.8–15.4)	25.5 (23.8–27.1)	
Severe	2.9 (2.5–3.3)	1.9 (1.5–2.2)	7.2 (6.1–8.2)	
**Body mass index, kg/m^2^ c**				
<19 (underweight)	2.5 (2.2–2.9)	2.4 (2.0–2.8)	3.0 (2.3–3.7)	<.001
≥19 and <25 (normal weight)	28.9 (27.6–30.26)	29.6 (28.0–31.1)	26.2 (24.5–28.0)	
≥25 and <30 (overweight)	33.1 (32.1–34.1)	33.6 (32.4–34.7)	30.9 (29.0–32.9)	
≥30 (obese)	34.2 (32.9–35.5)	33.2 (31.6–34.8)	38.4 (36.5–40.2)	
**Smoker^d^**				
Never	53.1 (51.4–54.9)	54.9 (53.0–56.8)	45.8 (42.7–48.8)	<.001
Former	22.1 (21.0–23.2)	23.8 (22.6–25.1)	14.7 (13.0–16.3)	
Current	22.4 (21.1–23.7)	18.9 (17.7–20.2)	36.9 (34.0–39.8)	
**Alcohol consumption^e^**				
None or moderate	92.5 (91.8–93.2)	92.6 (91.7–93.4)	92.1 (91.1–93.1)	.42
Risky	7.5 (6.8–8.3)	7.5 (6.6–8.3)	7.9 (6.9–8.9)	
**No insurance**	19.4 (17.9–20.9)	14.3 (13.0–15.5)	40.7 (38.4–43.0)	<.001
**No usual care**	16.7 (15.6–17.7)	13.4 (12.4–14.3)	30.6 (28.2–32.9)	<.001

Abbreviation: CI, confidence interval.

a Values are expressed as percentage (95% CI), unless otherwise indicated. All values reflect point estimates (95% CI) using survey weights to provide a nationally representative estimate.

b *P* values calculated using Rao Scott χ^2^ tests (for categorical variables) or *F* test (for continuous variables).

c Measurements were assessed at a mobile examination center and represent a subsample of the total.

d Current smoker was define as currently smoking or quit within the past 12 months, former as quit ≥12 months ago, never smoked as a lifetime history of smoking ≤100 cigarettes.

e Risky was defined a >1 drink/d for women or >2 drinks/d for men and moderate as ≤1 drink/d for women and ≤2 drinks/d for men.

We measured health correlates in 3 broad areas associated with food security and food assistance: general health and functional status, health risk behaviors, and health care access indicators (2,4,5,9,17–21). Health characteristics — except body mass index (BMI, kg/m^2^), which was measured directly — were self-reported.

For general health and functional status, we considered self-rated health, which we defined as excellent, very good, or good health versus fair or poor. Poor physical and poor mental health days were defined as the number of days (0–30) in the last month in which physical or mental health was self-reported as not good. BMI was defined as underweight (<19), normal weight (19–24), overweight (25–29), or obese (≥30). Receipt of a diagnosis of arthritis, cancer, diabetes, or hypertension was defined by yes/no answers. Experiencing depressive symptoms in the last 2 weeks was classified by using the Patient Health Questionnaire (PHQ-9) scores as severe (≥14), mild/moderate (≥5 to ≤13) or none/minimal (≤4) (22). Functional limitations (19 questions) were present if respondents reported “some” or “much” difficulty or “unable to do” for 1 or more activities in each of 5 functional groups: lower extremity mobility, general physical activity, activities of daily living, instrumental activities of daily living, and leisure and social activities (23).

Smoking and alcohol consumption were assessed as health risk behaviors. Smoking was measured as current smoker (currently smoking or quit within the past 12 months), former smoker (quit ≥12 months ago), or never smoked (a lifetime history of smoking ≤100 cigarettes). Alcohol use was defined as risky drinking (>1 drink/d for women or >2 drinks/d for men) or moderate or no drinking (≤1 drink/d for women and ≤2 drinks/d for men).

Health care access indicators, assessed with a yes or no answer, were currently having health insurance coverage and currently having a usual source of care (a place where a person usually goes when sick or in need of health-related advice).

### Statistical analysis

Sample weights were used to generate nationally representative estimates for the noninstitutionalized civilian population of US adults aged 20 years or older. Interview weights were used for all variables except those collected at the mobile examination center (BMI, self-rated health, number of poor physical and mental days) wherein examination sample weights were used. Weights were divided by number of combined surveys to estimate population average (23,24).

We characterized correlates of food security (insecure vs secure) and receipt of food assistance (no assistance, SNAP only, charity only, or both SNAP and charity) among people who were food insecure by using weighted prevalence estimates with associated 95% confidence intervals (CIs) and *P* values using Rao Scott χ^2^ or *F* test (2-sided tests, significance set at *P* ≤ .05). We used SAS version 9.4 (SAS Institute Inc) SURVEY procedures to incorporate the NHANES complex sampling design. Last, we examined multivariate correlates of food assistance receipt by using a multinomial model accounting for interview weights using the SVY command in Stata/SE version 14.1 (StataCorp, LP). For the multivariate model, we simplified covariates as follows: education (<high school diploma, high school diploma, some college/college degree, or more/other), poverty–income ratio (continuous), physical function limitations (any vs none), number (0, 1, ≥2) of comorbid conditions (arthritis, diabetes, or hypertension), depression symptoms (mild/severe vs none), and BMI (continuous). We dropped household size, because it was highly correlated with having household children and dropped cancer, insurance, and usual care because they were not associated with food assistance in univariate analysis. Coefficients of these multinomial models were exponentiated to obtain *relative risk ratios* and their 95% confidence intervals (CIs), which can be interpreted in the same way as an odds ratio, albeit with multiple response categories. The relative risk ratio reflects the difference for a 1-unit increase in the value of any covariate *k* of being in category *j *= 2, 3, 4 (receives SNAP only, charity only, or both) relative to category *j* =1 (receives no food assistance), given that the other covariates are held constant. 

## Results

Of the 16,934 eligible adults in our sample, 4,555 lived in food insecure households, representing 19.3% of the US population (95% CI, 17.9%–20.7%). Of people who were food insecure, 58.0% (95% CI, 54.5%–61.6%) received no food assistance, 20.3% (95% CI, 18.0%–22.6%) received only SNAP benefits, 9.7% (95% CI, 8.2%–11.1%) received only charity food (eg, food bank), and 12.0% (95% CI, 10.0%–14.0%) received both SNAP benefits and charity food.

All sociodemographic characteristics differed by food security versus insecurity. Compared with food-secure respondents, people who were food insecure were more likely to be younger, female, Hispanic, non-Hispanic black; unmarried, less educated (less than a high school diploma); and were more likely to live in larger and higher poverty households (smaller poverty-to-income ratio) and households with children (Table 1).

Compared with food-secure respondents, people who were food insecure had poorer self-rated health, more frequent poor physical and mental health days, higher BMI, and higher prevalence of diabetes, smoking, depressive symptoms, and every type of functional limitation (Table 1). Prevalence of severe depressive symptoms was almost 4 times higher among people who were food insecure than among those who were not. People who were food insecure were 2 to 3 times more likely to have limited health care access (ie, no insurance or no usual care). In contrast, alcohol consumption did not differ across groups, and arthritis, cancer, and hypertension were less common among people who were food insecure.

Among people who were food insecure, all sociodemographic characteristics differed by participation in a food assistance program (Table 2). In general, participants receiving SNAP alone or both SNAP and charity assistance were more similar to each other than to those receiving charity food alone or no assistance. Among people who were food insecure, all health characteristics and behaviors, excepting cancer history, differed by program participation. In general, respondents receiving both SNAP and charity assistance had the worst health while respondents who received no assistance had the best health.

**Table 2:** Sociodemographic and Health Characteristics of Food Insecure Participants (N = 4,555), Stratified by Food Assistance Program Participation,, National Health and Nutrition Examination Survey, 2005–2010^a^

Characteristic	Type of Assistance Program				
	None, N = 2,498	Charity Only, N = 473	SNAP Only, N = 993	Both, N = 591	*P* Value^b^
Sociodemographics					
**Age, mean (95% CI)**	41.5 (40.6–42.5)	41.9 (39.8–43.9)	39.4 (38.4–40.4)	41.3 (40.1–42.5)	.004
**Sex**					
Male	48.6 (46.5–50.6)	45.8 (41.6–50.0)	43.1 (40.5–45.7)	39.9 (37.1–42.7)	<.001
Female	51.5 (49.4–53.5)	54.2 (50.0–58.4)	56.9 (54.3–59.5)	60.1 (57.3–62.9)	
**Race/ethnicity**					
Non-Hispanic white	45.9 (39.2–52.6)	48.5 (38.7–58.3)	43.3 (33.1–53.4)	49.2 (40.3–58.1)	.01
Hispanic	29.9 (24.2–35.6)	26.2 (18.7–33.6)	28.9 (21.0–36.8)	19.4 (13.1–25.7)	
Non-Hispanic black	17.013.4–20.7)	20.1 (14.3–25.9)	22.3 (16.0–28.7)	27.9 (21.4–34.4)	
Other	7.2 (4.8–9.6)	5.2 (2.5–8.0)	5.5 (3.0–8.0)	3.5 (1.6–5.4)	
**Marital status**					
Married	57.3 (54.2–60.3)	49.4 (44.5–54.3)	51.6 (46.2–57.0)	42.6 (38.3–46.8)	<.001
Single	21.3 (18.8–23.7)	23.8 (18.7–28.8)	24.9 (20.5–29.2)	27.1 (23.0–31.3)	
Widowed/Separated/ Divorced	21.5 (19.2–23.7)	26.9 (21.7–32.1)	23.5 (20.1–27.0)	30.3 (26.1–34.5)	
**Education**					
Less than high school	31.6 (28.5–34.8)	38.8 (32.6–45.0)	44.7 (40.0–49.4)	42.3 (37.2–47.3)	<.001
High school graduate	27.9 (25.5–30.4)	25.3 (21.2–29.4)	29.0 (25.5–32.6)	29.3 (24.8–33.7)	
Some college, college degree, or more/other	40.4 (37.1–43.8)	36.0 (30.3–41.6)	25.9 (21.6–30.1)	28.0 (22.8–33.2)	
**Poverty-to-income ratio**					
<1	26.5 (22.9–30.1)	40.5 (32.4–48.5)	61.2 (55.1–67.2)	62.3(55.2–69.5)	<.001
≥1 to <1.33	13.7 (11.7–15.6)	19.5 (14.5–24.5)	18.3 (14.4–22.1)	16.6 (13.2–20.0)	
≥1.33 to < 1.50	6.8 (5.4–8.3)	7.0 (4.3–9.7)	3.4 (2.2–4.7)	6.6 (3.0–10.2)	
≥1.50 to <1.85	10.9 (8.9–12.9)	12.4 (8.1–16.8)	4.4 (2.7–6.1)	5.3 (2.6–7.9)	
≥1.85	42.1 (37.9–46.4)	20.6 (15.2–26.1)	12.7 (8.6–16.9)	9.2 (5.3–13.1)	
**Household size**					
1 or 2	35.8 (31.6–40.1)	38.1 (31.1–45.0)	24.2 (20.4–27.9)	32.5 (26.0–39.0)	<.001
3 or 4	38.6 (35.0–42.3)	38.7 (30.7–46.7)	36.4 (31.6–41.2)	38.5 (32.4–44.6)	
≥5	25.6 (21.3–29.8)	23. 3(17.3–29.2)	39.5 (33.5–45.5)	28.9 (23.2–34.7)	
**Children in home aged 18 y or younger**	52.1 (47.9–56.3)	49.7 (41.9–57.4)	71.5 (66.8–76.3)	58.0 (53.0–63.0)	<.001
**Health**					
**No. of days per month^c^**					
Poor physical health	4.3 (3.8–4.8)	5.6 (4.4–6.9)	5.8 (4.8–6.8)	7.3 (6.3–8.2)	<.001
Poor mental health	5.3 (4.8–5.8)	7.8 (6.4–9.3)	7.4 (6.7–8.1)	10.2 (9.1–11.4)	<.001
**Self-rated health^c^**					
Excellent/very good/good	66.6 (64.0–69.1)	61.7 (55.1–68.2)	57.7 (53.5–61.8)	52.5 (47.3–57.7)	<.001
Fair/poor	23.6 (21.3–25.9)	26.1 (21.8–30.5)	31.4 (27.2–35.5)	40.4 (35.1–45.6)	
**Functional limitations**					
Any	25.5 (23.3–27.6)	38.3 (32.2–44.3)	35.6 (31.6–39.6)	52.9 (47.7–58.2)	<.001
Activities of daily living	11.4 (9.9–12.9)	21.9 (17.2–26.7)	17.8 (14.4–21.1)	33.7 (29.1–38.3)	
Instrumental activities of daily living	13.9 (12.2–15.5)	24.7 (18.8–30.6)	21.3 (18.1–24.5)	36.7 (31.9–41.4)	
Lower extremity mobility	8.2 (6.9–9.5)	13.5 (9.6–17.4)	14.1 (11.2–16.9)	20.2 (16.5–23.8)	
General physical activity	23.9 (21.7–26.1)	35.0 (29.3–40.8)	32.7 (28.9–36.5)	49.6 (44.5–54.7)	
Leisure and social activities	11.1 (9.5–12.8)	22.4 (18.3–26.5)	17.4 (14.6–20.2)	32.2 (28.3–36.2)	
**Comorbid conditions**					
Arthritis	19.7 (17.4–22.0)	25.0 (20.7–29.3)	22.7 (19.7–25.7)	37.8 (33.0–42.6)	<.001
Cancer	5.6 (4.4–6.8)	6.0 (4.0–8.0)	4.3 (2.9–5.8)	7.0 (4.9–9.1)	.19
Diabetes	8.5 (7.3–9.8)	9.1 (6.2–12.0)	9.3 (7.2–11.4)	12.7 (9.7–15.8)	.045
Hypertension	26.1 (24.0–28.2)	31.5 (26.1–36.9)	27.6 (24.3–30.8)	35.0 (29.4–40.7)	.006
**Depressive symptoms in the last 2 weeks**					
None	72.4 (69.8–75.0)	67.1 (60.8–73.4)	62.9 (59.5–66.2)	50.9 (47.0–54.8)	<.001
Mild	22.5 (20.3–24.7)	23.6 (18.6–28.5)	29.5 (26.1–32.9)	34.5 (30.2–38.9)	
Severe	5.1 (3.8–6.4)	9.4 (6.2–12.5)	7.6 (5.6–9.6)	14.6 (11.5–17.7)	
**Body mass index, kg/m^2^ c ^,^ d**					
<25 (normal weight or underweight)	30.1 (27.7–32.5)	24.7 (17.9–31.6)	30.8 (27.1–34.5)	26.4 (22.3–30.5)	.03
≥25 to <30 (overweight)	31.8 (29.4–34.3)	33.1 (28.0–38.3)	29.9 (26.6–33.3)	26.3 (22.5–30.2)	
≥30 (obese)	36.7 (34.1–39.4)	41.4 (35.3–47.6)	37.4 (34.0–40.8)	45.2 (40.6–49.8)	
**Smoker^e^**					
Never	51.8 (48.3–55.4)	40.7 (34.5–46.8)	40.8 (35.1–46.5)	29.0 (23.7–34.3)	<.001
Former	16.2 (14.2–18.2)	13.5 (8.6–18.4)	12.6 (9.9–15.3)	11.5 (9.0–14.0)	
Current	29.1 (25.8–32.5)	44.2 (36.0–52.4)	44.2 (38.3–50.1)	56.7 (51.2–62.1)	
**Alcohol consumption**					
None or moderate^f^	92.6 (91.4–93.7)	95.3 (93.0–97.7)	91.8 (89.3–94.4)	87.4 (84.3–90.6)	.001
Risky	7.4 (6.3–8.6)	4.7 (2.3–7.0)	8.2 (5.6–10.7)	12.6 (9.4–15.7)	
**No insurance**	39.5 (35.9–43.1)	45.3 (39.5–51.1)	42.7 (38.5–46.8)	39.6 (33.8–45.3)	.34
**No usual care**	30.4 (27.3–33.5)	32.5 (27.7–37.3)	31.1 (27.2–35.0)	28.8 (25.1–32.6)	.71

Abbreviations: CI, confidence interval; SNAP, Supplemental Nutrition Assistance Program.

a Values are expressed as % (95% CI), unless otherwise indicated. All values reflect point estimates (95% CI) using survey weights to provide a nationally representative estimate.

b *P* values calculated by Rao Scott χ^2^ tests (for categorical variables) or *F *test (for continuous variables).

c Measurements assessed at mobile examination center represent a subsample of the total.

d Underweight and normal weight individuals were collapsed due to small cell sizes across categories of food assistance.

e Current smoker was define as currently smoking or quit within the past 12 months, former as quit ≥12 months ago, never smoked as a lifetime history of smoking ≤100 cigarettes.

f Risky was defined a >1 drink/d for women or >2 drinks/d for men and moderate as ≤1 drink/d for women and ≤2 drinks/d for men.

Sociodemographic and health characteristics differed by program participation in multivariable analyses (Table 3). People in higher poverty households (smaller poverty-to-income ratio) and current smokers were more likely to receive any type of assistance (charity only, SNAP only, or both) versus no assistance. People with more poor mental health days were more likely to receive charity only or both types of assistance versus no assistance. People with 2 or more comorbid conditions; those widowed, divorced, or separated; and those with children in the home were more likely to receive SNAP only or both types of assistance versus no assistance. Additional health and sociodemographic characteristics more common among those receiving both types of assistance versus no assistance are listed in Table 3.

**Table 3:** Multivariate Multinomial Regression for the Association Between Sociodemographic and Health Characteristics and Food Assistance Program Participation Among People Who Are Food Insecure, National Health and Nutrition Examination Survey, 2005–2010^a^

Characteristic	Food Assistance Program Participation^b^		
	SNAP Only	Charity Only	Both
	Relative Risk Ratio (95% Confidence Interval)		
**Age, y**	0.99 (0.98–1.00)	1.00 (0.98–1.01)	0.98 (0.97–1.00)
**Sex**			
Male	Reference [1]	Reference [1]	Reference [1]
Female	0.98 (0.84–1.14)	0.99 (0.81–1.21)	1.03 (0.84–1.26)
**Race/ethnicity **			
Non-Hispanic white	Reference [1]	Reference [1]	Reference [1]
Hispanic	0.65 (0.40–1.06)	0.96 (0.62–1.48)	0.51 (0.31–0.86)
Non-Hispanic black	1.21 (0.74–1.97)	1.22 (0.75–2.00)	1.44 (0.87–2.38)
Other	0.88 (0.38–2.07)	0.95 (0.45–1.99)	0.69 (0.33–1.44)
**Marital status**			
Married	Reference [1]	Reference [1]	Reference [1]
Single	1.39 (0.96–2.02)	0.97 (0.67–1.39)	1.41 (0.93–2.14)
Widowed/separated/divorced	1.59 (1.18–2.14)	1.31 (0.90–1.91)	1.55 (1.13–2.14)
**Education**			
Less than high school	Reference [1]	Reference [1]	Reference [1]
High school diploma	1.01 (0.75–1.36)	0.81 (0.59–1.11)	0.86 (0.63–1.19)
Some college, college degree, or more/other	0.65 (0.41–1.01)	0.91 (0.62–1.33)	0.64 (0.43–0.96)
**Poverty-to-income ratio (larger ratio = higher income/lower poverty)**	0.23 (0.16–0.33)	0.46 (0.33–0.64)	0.17 (0.10–0.29)
**Children in home aged 18 y or younger**			
No	Reference [1]	Reference [1]	Reference [1]
Yes	2.76 (1.94–3.94)	1.02 (0.61–1.71)	1.93 (1.33–2.80)
**No. of days per month**			
Poor physical health	1.00 (0.99–1.02)	1.00 (0.98–1.02)	1.00 (0.98–1.01)
Poor mental health	1.01 (1.00–1.02)	1.02 (1.01–1.04)	1.02 (1.01–1.04)
**Self-rated health**			
Excellent/very good/good	Reference [1]	Reference [1]	Reference [1]
Fair/poor	0.98 (0.75–1.29)	1.14 (0.85–1.54)	0.88 (0.66–1.16)
**Functional limitations **			
None	Reference [1]	Reference [1]	Reference [1]
Any	1.27 (0.92–1.77)	1.18 (0.86–1.61)	2.24 (1.37–3.67)
**No. comorbid conditions^c^**			
0	Reference [1]	Reference [1]	Reference [1]
1	1.14 (0.79–1.66)	1.03 (0.74–1.42)	1.35 (0.97–1.88)
≥2	1.48 (1.04–2.11)	0.97 (0.58–1.60)	1.81 (1.10–2.96)
**Depressive symptoms in the last 2 weeks**			
None	Reference [1]	Reference [1]	Reference [1]
Mild/severe	1.18 (0.91–1.53)	0.97 (0.67–1.39)	1.22 (0.88–1.68)
**Body mass index, kg/m^2^**	1.00 (0.99–1.02)	1.01 (0.99–1.04)	1.02 (1.00–1.03)
**Alcohol consumption**			
None/moderate	Reference [1]	Reference [1]	Reference [1]
Risky	1.01 (0.59–1.71)	0.53 (0.28–1.01)	1.53 (1.01–2.32)
**Current smoker**			
Never/former	Reference [1]	Reference [1]	Reference [1]
Current	1.68 (1.17–2.42)	2.12 (1.34–3.35)	2.66 (2.11–3.34)
**Constant**	0.07 (0.02–0.24)	0.05 (0.02–0.17)	0.02 (0.01–0.08)

Abbreviations: BMI, body mass index; CI, confidence interval; RRR, relative risk ratio.

a Model was fitted using survey weights to provide a nationally representative estimate.

b Reference (base) category is no receipt of food assistance.

c Comorbid conditions assessed were arthritis, diabetes, hypertension.

## Discussion

Analyzing nationally representative data, we found that people who were food insecure disproportionately experienced adverse health across several dimensions. Results confirm previous observations of significant health disparities among the food insecure (2–5,17,19,20). For example, among people who are food insecure, compared with those who are not, we noted disproportionately poorer health and functional status, higher prevalence of risky health behaviors, greater prevalence of mild and severe depressive symptoms, and limited access to health care.

In contrast, people who are food insecure were less likely to report arthritis, cancer, and hypertension. In light of their limited health care access, this may not indicate a health advantage but rather fewer opportunities to obtain nonacute medical care. Consistent with this interpretation, previous research indicated unmet needs for chronic disease screening and prevention among people who were food insecure (7).

In univariate analysis, respondents receiving both SNAP and charity food experienced the poorest health and functional status. In contrast, we found that those receiving no assistance had the best health and functional status. Surprisingly, health care access measures did not differ by program participation. One might assume that an individual who successfully navigated a food assistance program would be more likely to have engaged with safety-net health care systems (eg, Medicaid, a federally qualified health center). Because health care access did not differ by program participation, health differences may be a result of other factors, such as access to transportation, preventive health behaviors, or cultural norms related to care-seeking behavior.

In multivariable analysis, several variables were associated with receipt of food assistance. Overall, 2 factors were consistently associated with receipt of any assistance: those receiving assistance of any kind (vs those receiving none) were more likely to be smokers and live in higher poverty households. Multiple factors were associated with receiving SNAP only and receiving both types of assistance, including being widowed, separated, or divorced; having children in the home; and having 2 or more comorbid conditions. Although we cannot ascertain which type of assistance was sought out first, this finding may indicate that these populations seek food first from SNAP and later, as needed, supplement SNAP with additional charity food.

We sought to identify relationships among food security, food assistance, and health without inferring causality, given our cross-sectional design. Without appreciation for complex relationships among these factors, one might interpret our findings by concluding that food assistance causes poor health. This seems highly unlikely given prior robust research indicating improved health and diet quality among food assistance recipients, particularly SNAP enrollees (18,24).

Alternative explanations may underlie associations between program participation and health. For example, poor health may function as a cause rather than an outcome of program participation. For example, a wage earner with a chronic illness becomes unemployed because of unmanageable symptoms, leading to lost income and food insecurity, at which point SNAP participation is initiated. Alternatively, program participation may be a proxy for severity of food insecurity or material hardship, citizenship status, availability of or access to food assistance, or previous patterns of food security or food assistance that we could not measure in this study. Longitudinal data are needed to understand the complex, potentially cyclical interrelationships between food insecurity, food assistance program participation, and health.

### Implications for targeted recruitment and outreach

Our findings have implications for identification and targeting of food insecure populations for future prevention research, intervention, and policy. For example, we found that those receiving both SNAP and food from charities experienced the worst health, particularly depressive symptoms and smoking. Thus health education and behavior change interventions may be particularly needed among this subpopulation.

The best approach to target food insecure subpopulations is unclear. We previously reported that waiting rooms in nonprofit food distribution centers are an ideal place to recruit food insecure individuals for clinical research (9). We reached this conclusion in part because of ease of recruitment, client willingness, and enthusiastic support of our food distribution center partner. These findings suggest that in addition to charity-only clients, this approach also captures the subpopulation with the poorest health (ie, those receiving SNAP and charity food). However, this approach cannot capture a representative sample of all people who are food insecure. To guide future research, we describe strategies that may facilitate primary data collection and participant recruitment of these populations.

### Non-probability and probability sampling

To date, most research has used nonprobability sampling. Various approaches can be taken for identifying food insecure individuals including recruitment via 1) privately funded food assistance programs (ie, food banks, distribution centers, pantries, meal programs) (7,9,11,12,25,26); 2) publicly funded government benefits programs (10,27); and 3) health care system settings (ie, federally qualified health centers) (Table 4) (3,19,28,29). For each, we describe potential for selection bias, practical considerations, and examples of nonprobability recruitment methods.

**Table 4:** Recruitment Strategies for Nonprobability Samples of Food Insecure Adult Populations in the Food Assistance and Health Care System Sectors

Example Nonprobability Sampling Frame	Food Assistance Sector		Health Care System
	Private (Charity)	Public	In-Person Recruitment at Waiting Room in Saftey-Net Health Care System (eg, Federally Qualified Health Center)
	In-Person Recruitment in Waiting Room of Staging Or Delivery Locations for Food Distribution Center, Food Pantry, Meal Program	In-Person Recruitment at Government Enrollment Offices (eg, SNAP)	
**Subpopulations included based on receipt of food assistance**			
SNAP only		x	x
Charity only	x		x
Both	x	x	x
None			x
**Concentration of food insecure**	High		Varied
**Selection bias**	May be biased toward current recipients of food assistance		Underrepresents individuals with limited health care access
**Advantages**	Potential to link with objectively measured eligibility and food assistance data		Potential to link with objectively measured health status and health care use data
	Potential for improved recruitment because of client trust of partner organization	—	Facilitates identification of food insecure with specific health needs, conditions
**Disadvantages**	Program catchment area may be small (eg, single metropolitan area) resulting in small geographic scope	Significant administrative, government approval processes; additional restrictions related to data release, sharing, storage, destruction	
		—	Costly because of time and effort required to screen patients to identify food insecure

Abbreviation: —, not applicable; SNAP, Supplemental Nutrition Assistance Program.

With the exception of secondary analyses of national survey data, probability sampling is rare. Approaches such as door-to-door screening or random-digit dialing are time intensive and cost-intensive (5,8). In contrast, programmatic and administrative databases represent a promising, feasible, and low-cost approach for probability sampling from within the food assistance sector. A sampling frame could be generated from such data to identify target populations based on client characteristics such as type or amount of food assistance received. This approach has several advantages, including generalizability and opportunity to capitalize on additional program data (eg, current and historical trends in client eligibility, use of services, amount of food or benefits received). Administrative data may also facilitate identification of former clients no longer receiving assistance. Inclusion of this population in future research is critical to understand the cyclical relationships between economic insecurity, food insecurity, food assistance, and health.

We presume that administrative databases exist within public food assistance programs. However, we are unaware of previous health studies using such data for probabilistic recruitment. In the private food assistance sector, database availability is less certain and is likely limited to larger, integrated food bank distribution systems, such Feeding America network members. Notably, existing administrative databases in public or private sectors may not be research-ready, requiring significant upfront investments. For example, data may exist in multiple and unlinked databases, codebooks may not exist, or historical data may be overwritten.

Identifying and sharing existing private sector administrative databases is one way to advance research related to health and food insecurity. For example, The Hunger Center Longitudinal Database (HCLD) is a research-ready database of food bank clients (30). As one example of its functionality, HCLD can support probabilistic stratified sampling of charity-only recipients and charity plus SNAP recipients. HCLD is the result of significant investments in infrastructure, data sharing agreements, personnel, and the combined contributions of academic researchers and stakeholders. Additional efforts are needed to develop similar resources across diverse private programs.

Future work is needed to develop and deploy recruitment methods with which to identify and target representative samples of food-insecure populations. By producing more generalizable research, we can facilitate future policies and programs designed to ensure health equity for all food insecure populations.

Although recruitment and outreach are important, further work is needed to develop, implement, and evaluate interventions designed to improve the health of people who are food insecure. Additional intervention development and evaluation research is needed because this population’s response to various intervention strategies may differ from that of other vulnerable populations in unknown ways. Recently, an intervention study found differential effects by client food security status. A study of home blood pressure telemonitoring and telephone-based case management of men and women with type 2 diabetes and uncontrolled hypertension found that although food secure participants achieved a clinically and significant reduction in systolic blood pressure, no change occurred among food insecure participants (29). All were low-income and either African American or Hispanic, and results did not change after adjustment for income. These results, although preliminary, suggest that traditional intervention strategies may need refinement before implementation among people who are food insecure. Furthermore, as indicated by our findings, intervention strategies may need further targeting for people who differ by food assistance program participation. As a first step, however, food assistance program staff could routinely inform clients of affordable, local health resources, such as safety-net health clinics and community mental health centers.

Our study has several limitations. First, we sought to describe differences in subpopulations of the food insecure to inform future recruitment and outreach efforts and not to assess causality between food assistance and health. Future prospective research should assess antecedents and subsequent effects of food insecurity. Second, we could not measure informal food assistance or frequency of assistance, which may influence health. Third, given eligibility criteria, which would have dramatically reduced our sample, we did not assess WIC participation. Fourth, we measured food insecurity in the year before the survey and could not assess its chronicity or its severity. Finally, program eligibility varies geographically; however, NHANES does not allow geographic analysis.

Our study also has strengths. We analyzed nationally representative data and measured food security by using the gold standard measure (16). Although ours is not the first study to do so (1,17), it has unique advantages. For example, in contrast to existing disease- or behavior-specific literature, we provided a comprehensive picture of multiple health conditions, risk behaviors, and health care access factors associated with food insecurity. In doing so, we identified notable disparities related to self-rated health, current smoking, depressive symptoms, and health care accessibility. As another example, we used multivariate methods to examine how food assistance was associated with sociodemographic and health factors using. Our study is the first to identify heterogeneity within the food insecure population with respect to diverse health needs and challenges across populations receiving different types of food assistance. This novel finding provides an actionable leverage point from which future researchers and practitioners can enhance outreach to targeted subpopulations of people who are food insecure.
